# Characterization of Oral Microbiome and Exploration of Potential Biomarkers in Patients with Pancreatic Cancer

**DOI:** 10.1155/2020/4712498

**Published:** 2020-10-31

**Authors:** Haiyang Sun, Xia Zhao, Yanxia Zhou, Jun Wang, Rui Ma, Xi Ren, Huaizhi Wang, Lingyun Zou

**Affiliations:** ^1^Shenzhen Baoan Women's and Children's Hospital, Jinan University, 518102 Shenzhen, China; ^2^Bioinformatics Center, Department of Microbiology, Army Medical University, 400038 Chongqing, China; ^3^Institute of Hepatopancreatobiliary Surgery, Chongqing General Hospital, University of Chinese Academy of Sciences, China

## Abstract

Pancreatic cancer (PC) is highly malignant and lacks an effective therapeutic schedule, hence that early diagnosis is of great importance to achieve a good prognosis. Oral bacteria have been proved to be associated with pancreatic cancer, but the specific mechanism has not been comprehensively illustrated. In our study, thirty-seven saliva samples in total were collected with ten from PC patients, seventeen from benign pancreatic disease (BPD) patients, and ten from healthy controls (HC). The oral bacterial community of HC, PC, and BPD groups was profiled by 16S rDNA high-throughput sequencing and bioinformatic methods. As shown by Simpson, Inverse Simpson, Shannon and Heip, oral microbiome diversity of HC, BPD and PC groups is in increasing order with the BPD and PC groups significantly higher than the HC group. Principal coordinate analysis (PCoA) suggested that grouping by PC, BPD and HC was statistically significant. The linear discriminant analysis effect size (LEfSe) identified high concentrations of *Fusobacterium periodonticum* and low concentrations of *Neisseria mucosa* as specific risk factors for PC. Furthermore, predicted functions showed changes such as RNA processing and modification as well as the pathway of NOD-like receptor signaling occurred in both PC and HC groups. Conclusively, our findings have confirmed the destruction of oral bacterial community balance among patients with PC and BPD and indicated the potential of *Fusobacterium periodonticum* and *Neisseria mucosa* as diagnostic biomarkers of PC.

## 1. Introduction

Pancreatic cancer (PC) is a common malignant tumor, leading to around 432,242 deaths worldwide in 2018 [1]. Patients in the early stage of PC have few symptoms, and the disease is commonly diagnosed during intermediate and advanced stages for which there is no effective clinical treatment [2]. The five-year relative survival rate of PC patients was around 9% [3]. Therefore, looking for new potential biomarkers will benefit PC patient's early diagnosis greatly. CA19.9 (carbohydrate antigen 19.9) was released into blood by PC cells and widely used in clinical diagnosis [4, 5]. However, the testing result based on CA19.9, as well as other serological markers such as CEA (carcinoembryonic antigen) are not so reliable [6–8].

Lots of diseases were related to the imbalance in the body's microbiome [4, 9]. Actually, the oral cavity is one of the regions with the highest microbiome density and the largest number of species in the body [10]. Many studies have demonstrated that oral bacteria play an important role in the development of cancer, especially the tumors of oral and gastrointestinal tract [11]. For example, *Fusobacterium nucleatum* and *Porphyromonas gingivalis* have been frequently researched and discussed [12–14]. The oral microbes can communicate with other tissues of the digestive system through blood circulation, lymphokines, or biliary conduction [15], and oral bacteria were usually associated with cancer of the entire digestive system [16]. Previous 16S sequencing studies revealed the crucial role of oral bacteria in the development of pancreatic cancer, and several potential bacterial biomarkers were identified including *Aggregatibacter actinomycetemcomitans*, *Neisseria elongata*, *Porphyromonas gingivalis*, and *Streptococcus mitis* [17–19]. It can thus be seen that the community composition of oral microbes provided clues for early diagnosis, monitoring, prevention, and treatment of PC.

The serological markers are generally secreted into pancreatic juice or blood via pancreatic epithelial tissue cells, hence sampling is inconvenient. However, sampling saliva and test of it are relatively easy. Although several oral bacteria are found to be related with PC, the association between oral bacteria and PC as well as BPD and the pathogenic mechanism were not explored sufficiently [12, 14]. Under such circumstances, we carried out this research to discover new available salivary biomarkers of PC, and to comprehensively explain the potential mechanism of oral microbes in the pathogenesis of PC. Thirty-seven saliva samples were collected from ten PC patients, seventeen BPD patients, and ten HC subjects. Based on 16S rDNA sequencing results, we compared the microbial community among the three groups and demonstrated that microbiome diversity in both the BPD and PC groups was greatly higher than that in the HC group. LEfSe outputs disclosed that *Fusobacterium periodonticum* and *Neisseria mucosa* may be the potential biomarkers of PC. Furtherly, the predicted bacterial gene functions as well as the enriched pathways showed the significant variance between PC and HC groups concerning RNA processing and modification and enriched pathways such as the pathway of NOD-like receptor signaling. Our findings found the association between oral bacteria and PC, and possible applications of oral bacteria in diagnostic and treatment strategies for PC.

## 2. Materials and Methods

### 2.1. Subjects and Sample Collection

A total of ten PC patients, seventeen BPD patients and ten HC subjects were enrolled in this study. Exclusion criteria included antibiotic treatment within 8 weeks, oral disease, digestive disease, genetic disease, other cancers and immune system diseases at the time of sample collection. Both PC and BPD groups were diagnosed by endoscopic ultrasonography and histopathologic examination at the First Hospital Affiliated to Army Medical University, China. It should be noted that the BPD group includes pancreatitis, chronic pancreatitis, and benign pancreatic tumors. This study was approved by the Ethics Board at the Army Medical University and performed in accordance with the Helsinki Declaration and Rules of Good Clinical Practice. Written informed consent upon enrollment as well as questionnaires addressing past medical history and lifestyles was obtained from all the volunteers.

In order to avoid the medical intervention that provokes an alteration in the oral microbiome, the samples were taken from each enrolled subject as close as possible to the time of enrolment. The volunteers were asked not to brush their teeth the night before and the morning of sampling, and not to have breakfast. At eight a.m., the volunteers gargled their mouths with sterile saline before sampling, then held the natural, unstimulated saliva in their mouths for about 1 minute before slowly spitting it into the samplers. For each volunteer, 10 mL of saliva was collected and transferred to the cryopreservation box immediately, then it was returned to the laboratory for dispensing and numbering, and frozen at -80°C within 1 hour. Samples mixed with blood are not retained.

### 2.2. DNA Isolation, PCR, and Sequencing

The bacterial DNA was extracted from the salivary samples using the FastDNA® SPIN Kit for Soil (Majorbio, Shanghai, China) following the manufacturer's protocol, and DNAs were quantified via NanoDrop 2000. The V3–V4 region of the bacterial 16S ribosomal RNA (rRNA) gene, which is generally considered the standard target, was PCR-amplified using universal primers 338F 5′-ACTCCTACGGGAGGCAGCAG-3′ and 806R 5′-GGACTACHVGGGTWTCTAAT-3′. Meanwhile, an eight-base sequence served as the barcode was added, which was unique to each sample. PCR reactions (20 *μ*L) contained 4 *μ*L of 5x FastPfu Buffer, 2 *μ*L of 2.5 mM dNTPs, 0.8 *μ*L of each primer (5 *μ*M), 0.4 *μ*L of TransStart FastPfu DNA Polymerase, 0.2 *μ*L BSA, 10 ng template DNA, and ddH_2_O. Thermal cycling consisted of the initial denaturation at 95°C for 2 min, followed by 25 cycles at 95°C for 30 s, 55°C for 30 s, 72°C for 45 s and a final extension at 72°C for 10 min. The construction of high-throughput sequencing libraries and pair-end sequencing was performed using the Illumina MiSeq platform according to the manufacturer's recommendations. Finally, all the raw reads were deposited into the NCBI Sequence Read Archive (SRA) database (Bioproject accession Number: SRP237984).

### 2.3. Bioinformatic and Statistical Analysis

Firstly, after the raw DNA fragments were generated, we merged the paired-end reads into a single sequence by means of using FLASH software v.1.2.10 (Fast Length Adjustment of SHort reads) [20]. Then, 16S rRNA OTUs (operational taxonomic units) were selected from the combined reads via QIIME toolkit v.1.9.1 (Quantitative Insights into Microbial Ecology) [21]. Note that sequences with 97% identity were assigned to the same OTU and the one with the highest frequencies was selected as the representative sequence. Thirdly, we used the RDP classifier v.2.2 (The Ribosomal Database Project) to annotate taxonomic information for each representative sequence [22]. Before performing the subsequent analysis, we used USEARCH 11 to randomly select an equal number of OTU sequences for all samples according to the minimum number of OTU sequences in samples. And next, based on the subordinate OTUs of different research groups, the corresponding alpha diversity was calculated on the indexes that include Simpson, Heip and Shannon. Note that Simpson employed here shows the negative correlation with bacterial diversity, and other indexes are positively correlated. The statistical differences of the calculated alpha diversity indexes of three groups were analyzed by a nonparametric factorial Kruskal-Wallis sum-rank test (K-W test) and post hoc Dunn's multiple comparison test (Dunn's test) [23]. Beta diversity distance between samples was calculated using principal coordinate analysis (PCoA) to explain the phylogenetic variation based on the weighted UniFrac values. Beta diversity comparisons were done using analysis of similarities via ANNOSIM and NMDS. LEfSe was employed to compare the relative abundance of bacterial taxa between the groups, and the results were profiled via cladograms and taxonomic bar charts [24]. Only the bacterial taxa with a linear discriminant analysis (LDA) score greater than a certain value and presented statistically significant (*P* value < 0.05) can be considered the meaningfully different taxa. KEGG pathways and COG (clusters of orthologous groups) functions were predicted by PICRUSt (phylogenetic investigation of communities by reconstruction of unobserved states) based on 16S rDNA sequencing data [25]. Correlation study shows that PICRUSt's prediction accuracy can even reach 0.95, and it is recognized as one of the most suitable function prediction tools [25]. The predicted KEGG and COG results of PICRUSt were compared between groups according to the sequence number of the corresponding results using the K-W test and Dunn's test. Statistical significance was accepted at *P* value < 0.05.

## 3. Results

### 3.1. Clinical Characteristics of the Participants

After applying rigorous inclusion and exclusion criteria, a total of ten PC patients, seventeen BPD patients and ten HC subjects were finally involved in this study. All participants were in the state of oral healthy. Table S1 detailed the clinical characteristics of volunteers. There are no significant differences between these groups in terms of gender, smoking and drinking (*P* value < 0.05). Tumor differentiation exhibited that ten PC patients were distributed in all the stages. The average age of PC or BPD patients is relatively close to the onset age of PC or BPD. Although the HC group has a lower average age, it is still appropriate for this study as the control considering the temporal stability of the oral microbiome.

### 3.2. Differences in Salivary Microbiota Diversity

**Figure 1:**
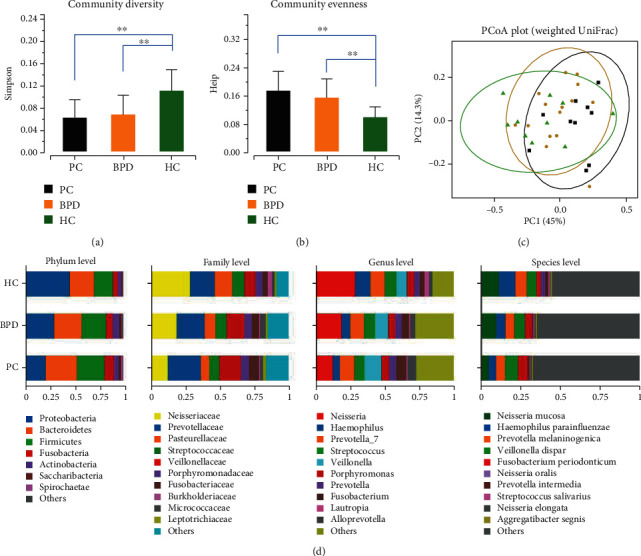
Phylogenetic diversity of oral bacteria community composition among the PC, BPD, and HC groups. (a) Histogram depicts community diversity according to the Simpson index among the PC, BPD, and HC groups. (b) Histogram depicts community evenness according to the Heip index among the PC, BPD, and HC groups. (c) Bacterial diversity clustering by weighted UniFrac PCoA of oral bacteria. Each symbol represents a sample and the variance explained by the PCs in parentheses on the axes. (d) Stacked bar depicts the relative proportions of oral bacteria among the PC, BPD, and HC groups at the phylum, family, genus, and species levels. Note: the symbol of “∗” represents the *P* value between groups which is less than 0.05.

To analyze the bacterial composition and differences among PC, BPD and HC groups, we applied high-throughput sequencing to detect the V3-V4 region of the bacterial 16S rRNA gene. As a result, 5,076,996 raw sequence reads with an average length of 445 bps were produced from all the thirty-seven samples. In total, 449 OTUs (operational taxonomic units) were obtained from the sequencing results, which include 19 phyla, 35 classes, 70 orders, 103 families, 193 genera, and 364 species. The Simpson and Heip indexes explicitly showed that the differences of oral bacterial community composition existed among the three groups. The mean value of the Simpson index was gradually decreased from the HC (0.113, SD = 0.039) to the BPD (0.067, SD = 0.023) and then the PC group (0.062, SD = 0.027) (Figure 1(a)). Both PC and BPD groups were significantly higher than the HC group, respectively (*P* value < 0.05), but there was no obvious difference between PC and BPD groups (*P* value > 0.05). The mean value of the Heip index was gradually increased from the HC (0.101, SD = 0.028) to the BPD (0.159, SD = 0.041) and then the PC group (0.178, SD = 0.048) (Figure 1(b)). The Heip index indicated that the PC group has the highest species uniformity followed by the BPD and HC groups. Moreover, the mean value of Inverse Simpson and Shannon indexes in the PC, BPD and HC groups was increased from the HC (10.016, SD = 3.880; 2.970, SD = 0.350) to the BPD (17.019, SD = 7.088; 3.514, SD = 0.393) and then the PC group (19.591, SD = 8.166; 3.587, SD = 0.430) (Figure S1).

As shown in Figure 1(c), the weighted UniFrac analysis depicted the distance relationship between samples of the PC, BPD, and HC groups. ANOSIM analysis showed that the classification of the three groups of samples yielded a *P* value of 0.02 and a *r* value of 0.12, which indicated that the among-group difference was greater than the intragroup difference. To further reveal the significance of grouping, we performed analysis of NMDS, and the results exhibited that the stress value of PC, BPD, and HC was 0.11 (less than 0.2). To sum up, the three groups in this study have statistically significant differences in the bacterial community structure, and the grouping of PC and HC is the most explanatory.

### 3.3. The Oral Bacterial Composition

As shown in Figure 1(d), the statistical analysis for bacterial abundance revealed the differences of the oral bacterium community structure among the PC, BPD, and HC groups. At the phylum level, the top five bacterial members of the PC, BPD, and HC groups were identical, including *Actinobacteria*, *Bacteroidetes*, *Firmicutes*, *Fusobacteria*, and *Proteobacteria*, and their proportion in total OTUs were 95.28%, 95.85%, and 98.06%, respectively. However, the relative proportions of dominant bacteria have changed. Both the dominant bacteria in the HC group (45.60%) and the BPD group (29.40%) were *Proteobacteria*. In contrast, the dominant bacteria in the PC group were *Bacteroidetes* (31.96%), and *Proteobacteria* was significantly reduced to 20.85%. At the family and genus levels, the dominant bacteria of the PC, BPD, and HC groups were identical, while the corresponded proportion was different from one another. The dominant bacteria in the HC group were *Neisseriaceae* (28.84%) at the family level and *Neisseria* (28.60%) at the genus level, while the dominant genus in the PC group was *Prevotellaceae* (23.72%), *Neisseria* (12.55%), and *Veillonella* (12.12%). The changing trend of dominant bacteria in the BPD group relative to the HC group was consistent with that of the PC group, but its magnitude was smaller than that of the PC group. At the species level, the PC, BPD, and HC groups possess the same top five bacteria, which are *Neisseria mucosa*, *Haemophilus parainfluenzae*, *Prevotella melaninogenica*, *Veillonella dispar*, and *Fusobacterium periodonticum*, but the relative proportions of dominant bacteria are different across the three groups. Both the dominant bacteria in the HC group (10.97%) and the BPD group (9.30%) were *Neisseria mucosa*. In comparison, the dominant bacteria in the PC group was *Veillonella dispar* (8.32%). Of the top five bacteria, *Neisseria mucosa* and *Fusobacterium periodonticum* showed great change amplitude across the HC (10.97%, 2.93%) and PC (4.60%, 5.94%) groups.

We carried out the K-W test and post hoc Dunn's test of the HC, BPD, and PC groups based on the OTUs at the phylum, family, and species levels. At the phylum level, two of nineteen bacterial taxa exhibited a significant difference between groups. The abundance of *Proteobacteria* in both the PC and BPD groups was notably lower as compared with the HC group, while the abundance of *Spirochaetae* in both the PC and BPD groups was markedly higher as compared with the HC group. At the family level, eight of one hundred and three bacterial taxa showed a significant difference between groups. *Neisseriaceae*, which was the dominant bacteria in the HC group, was dramatically lower in the PC group than both the HC and BPD groups. But *Prevotellaceae*, which was the dominant microbe in the PC group, displayed no distinct variation between groups. At the species level, thirty-six of three hundred and sixty-four bacterial taxa showed a distinct difference between groups. However, no statistically significant abundance difference of any bacterial taxa was detected between the PC and BPD groups.

### 3.4. Potential Saliva Biomarkers Associated with PC

**Figure 2:**
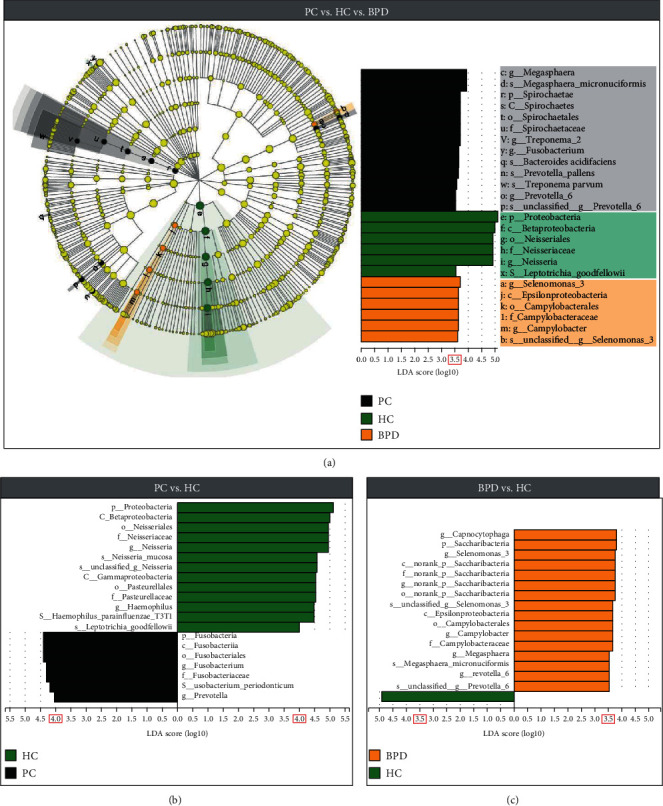
LEfSe and LDA analyses based on OTUs characterize bacteria among the PC, BPD, and HC groups. Cladogram using LEfSe strategy revealing the phylogenetic distribution of oral bacteria related to patients with PC (black), patients with BPD (orange), and HC subjects (green). LDA scores showed a significant bacterial difference between groups. The log10 scaled LDA score is indicated at the bottom, and the red box represents the selected threshold.

LEfSe was performed to further uncover the remarkable species of oral microbiota that characterizes the differences between the groups (Figure 2). Firstly, we exhibited the oral bacterial taxa between the HC, BPD, and PC groups (Figure 2(a)). When the log10 (LDA score) was greater than 3.5, the dominant bacteria in the PC group were *Fusobacterium*, *Megasphaera*, *Prevotella*, *Spirochaeta*, and *Treponema*, the dominant bacteria in the HC group were *Leptotrichia* and *Neisseria*, and the dominant bacterium in BPD was *Selenomonas*. We then explored the variant bacterial species between the PC and HC groups (Figure 2(b)). When the log10 (LDA score) was greater than 4.0, the dominant bacteria in the PC group were *Fusobacterium* and *Prevotella*, even including *Fusobacterium periodonticum* at the species level. Simultaneously, the dominant bacteria in the HC group were *Neisseria* and *Haemophilus*, even including *Neisseria mucosa*, *Haemophilus parainfluenzae*, and *Leptotrichia goodfellowii* at the species level. Finally, we compared bacterial species between the BPD and HC groups (Figure 2(c)). When the log10 (LDA score) was greater than 3.5, the dominant bacteria in the BPD group were *Campylobacter*, *Capnocytophaga*, *Megasphaera*, *Prevotella*, and *Selenomonas* at the genera level while the dominant bacterium in the HC group was only *Proteobacteria* at the phylum level.

### 3.5. Specific Changes of the Biological Functions

**Figure 3:**
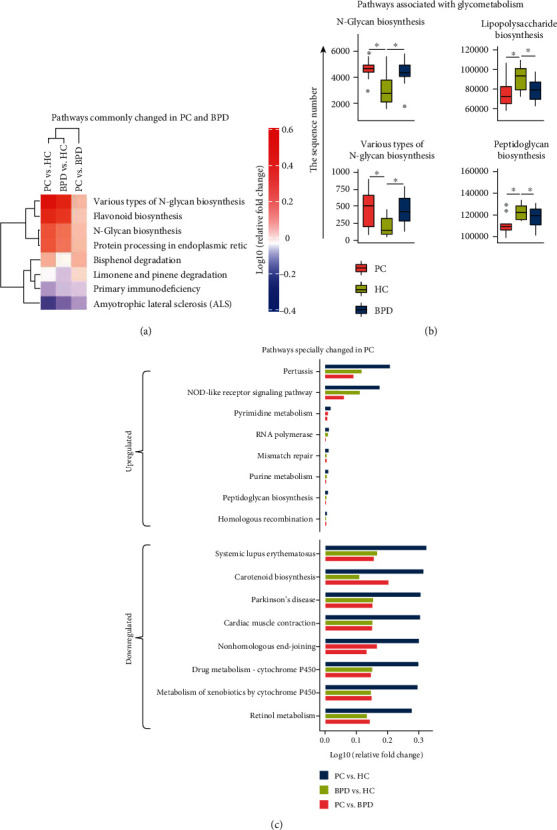
The KEGG pathway differences of oral bacteria among the PC, BPD, and HC groups. Statistical significance between groups was calculated by the K-W test and post hoc Dunn's test (*P* value < 0.05). (a) Heatmap displayed that eight KEGG categories were detected changed in both PC and BPD groups, and the log10 scaled relative fold change between the groups is indicated at the bottom. (b) Four pathways are associated with glycometabolism and were showed in the box plot based on the sequencing reads. The symbol of “∗” indicates that *P* value is less than 0.05. (c) Bar plots exhibited sixteen pathways specially changed in the PC group. Thereinto, eight categories are upregulated, whereas eight categories are downregulated.

**Figure 4:**
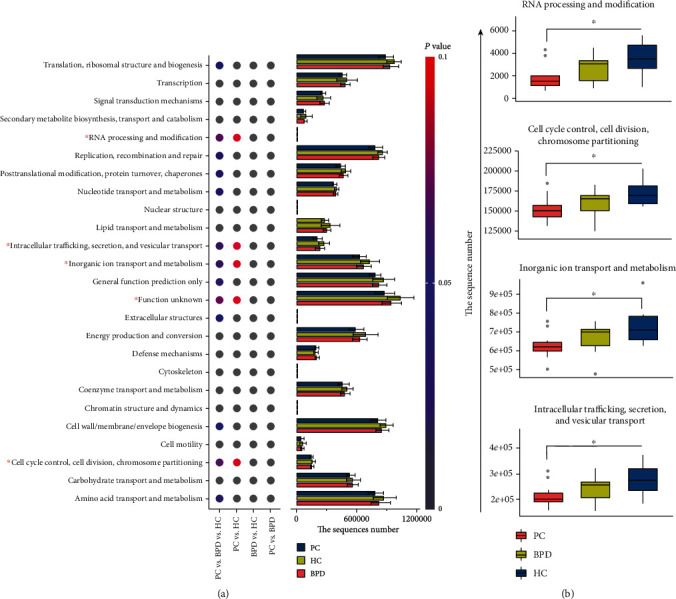
The COG function differences of oral bacteria among the PC, BPD, and HC groups. Statistical significance between groups was calculated by the K-W test and post hoc Dunn's test. (a) For the total twenty-five COG terms, the bar plot depicted the relative changes among the three groups based on the sequence number, and the dot plot exhibited the statistical significance between groups. (b) Box plots showed four COG functions apparently changed between PC and HC groups, which are observably or proven associated with pancreatic cancer. The symbol of “∗” indicates that *P* value is less than 0.05.

In order to characterize the changes in biological functions that may be involved in the PC, BPD, and HC groups, the PICRUSt toolkit was adopted to predict COG functions and KEGG pathways. As a result, a total of two hundred and eighteen KEGG pathways were predicted, and fifty-two were showed notably different between the three groups based on the number of sequences which are correlated with the pathways. Of the 52 KEGG pathways, 8 showed commonly significant changes in the comparison between the PC and HC groups as well as the BPD and HC groups (*P* value < 0.05), and Figure 3(a) portrayed the value of log10 relative fold change between groups for the eight pathways. Particularly, there were four of the fifty-two pathways involved in the glucose metabolism (Figure 3(b)), which are N-glycan biosynthesis, various types of N-glycan biosynthesis, lipopolysaccharide biosynthesis, and peptidoglycan biosynthesis. In addition, forty-two pathways showed significant differences between the PC and HC groups (*P* value < 0.05), of which eight pathways were upregulated and thirty-two pathways were downregulated. After that, an equal number of downregulated pathways were selected by the value of log10 relative fold change, and then, the fold change relationship between groups for the sixteen pathways is described in Figure 3(c). A total of twenty-five COG function terms were predicted, and only five terms exhibited significant difference between the three groups based on the number of sequences which are correlated with the COG terms. The twenty-five COG function terms may be divided into three categories, which are the cellular process and signaling, the information storage and processing, and the metabolism [26, 27]. In Figure 4(a), for each of the twenty-five COG function terms, the point diagram displayed the statistical relationships between groups, and the stacked bar conveyed the information about the number of correlated sequences of each group. The five COG function items that showed a significant difference between the three groups are “RNA processing and modification,” “cell cycle control, cell division, chromosome partitioning,” “inorganic ion transport and metabolism,” “intracellular trafficking, secretion, and vesicular transport,” and “function unknown.” Except for “function unknown,” Figure 4(b) portrayed the distribution of the number of sequences of all the samples in the three groups.

Not only does there exists a strong correlation of oral bacteria with pancreatic cancer, but we also presented here the relationship of oral microbiome with other cancers. Firstly, in contrast to healthy controls, patients with oral squamous cell carcinoma are usually carrying a higher proportion of bacteria of *Streptococcus anginosus*, *Streptococcus mitis*, *Capnocytophaga gingivalis*, *Prevotella melaninogenica*, and *Porphyromonas gingivalis* orally [28–30]. Then, oral mucosal cancer subjects have higher salivary counts of *Streptococcus intermedius*, *Streptococcus constellatus*, *Streptococcus oralis*, *Streptococcus mitis*, *Streptococcus sanguis*, and *Streptococcus salivarius*, which may be treated as the diagnostic indicators [31]. And next, head and neck squamous cell carcinoma also is associated with oral bacteria; prior studies showed the trend that the bacteria of *Streptococcus anginosus* were elevated [32]. Further, colorectal cancer patients with low levels of *Fusobacterium nucleatum* had a significantly longer overall survival time than patients with moderate and high levels of the bacterium [33]. In addition, *Streptococcus anginosus*, *Streptococcus mitis*, and *Treponema denticola* could have a significant role in the carcinogenic process of esophageal cancer by causing inflammation [34]. Finally, previous investigation described that *Oribacterium* sp. and *Fusobacterium sp.* could distinguish liver cancer patients from healthy subjects [35].

## 4. Discussion

PC has a high mortality rate, and the five-year survival rate of PC patients was about 9% [1]. PC is highly invasive with a poor prognosis and lacks effective therapeutic drugs [2]. Therefore, prevention at an early stage of PC is of crucial importance. As stated above, the oral cavity is characterized by the highest microbiome density and possesses the largest number of species in the body, and oral bacteria have been proved associated with many cancers, especially gastrointestinal tumors [10]. The architecture of microbiome communities orally seems to reflect health status in certain circumstances, making the analysis of oral microbiomes a promising approach for cancer diagnostics. Previous studies have shown that the imbalance of oral bacteria community composition was associated with the development of PC, and some bacteria may be the potential diagnostic markers [17–19]. The role of oral microbiota in the pathogenesis of cancer may be ascribed to the bacterial stimulation of chronic inflammation or secreting some virulence factors that act in a carcinogenic manner. Observably, the association of oral bacteria and PC as well as the BPD and the pathogenic mechanism were not yet explored sufficiently. This study is expected to contribute to this topic.

It is well known that species diversity increased with the rise of species richness as well as uniformity. Herein, we explored the species diversity of oral bacterial communities among the three groups. The performance of different groups on the Simpson uncovered that oral bacteria species diversity of both the PC group and the BPD group was obviously higher than that of the HC group, but there was no significant difference between PC and BPD. Other species diversity indexes such as Shannon also supported the result and correspond with the previous study [36]. In general, our findings demonstrated striking changes in the composition of the oral bacterium community among the PC and BPD groups as compared to the HC group. The differences perhaps related indirectly to the biological functions of the pancreas because of the imbalance in the body's microbiome [18, 36].

Discovering biomarkers has proven to be the most important and successful way to translate molecular and genomic research output into clinical applications [24]. Our efforts systematically shed light on the differences of oral bacterial community composition among the PC, BPD, and HC groups. While compared with the salivary bacterial community of the HC group, the PC group performed the consistent changing trend with the BPD group, but to a larger extent than the BPD group. At the phylum level, PC patients tended to have higher percentages of Bacteroidetes, Firmicutes, and Fusobacteria and lower percentages of Proteobacteria. At the finer taxonomic levels, we observed differences in the abundances of particular genera in PC patients compared with the HC and BPD groups. For example, the abundance of *Proteobacteria* and its subordinate *Neisseriaceae* and *Neisseria* was sharply reduced in the PC group, and the vantage of dominant bacteria was destroyed. Meanwhile, the ratio of *Fusobacteria* and its subordinate *Fusobacteriaceae*, *Fusobacterium*, and even the *Fusobacterium periodonticum* is greatly increased in the PC group.

LEfSe analysis further revealed the differences of the microbial community between the research groups. The greater the LDA score is, the more significant the microbial biomarker is in the comparison.  Figure 2 depicts the LEfSe results. In Figure 2(b), *Fusobacterium periodonticum* was specifically abundant in PC patients, meanwhile *Neisseria mucosa*, *Leptotrichia goodfellowii*, and *Haemophilus parainfluenzae T3T1* were characteristically enriched in the HC group. However, the population of *Leptotrichia goodfellowii* in the groups is very low (<0.002%), thus it is not appropriate to treat it as the candidate risk factor for recognizing PC patients from healthy individuals. As for *Haemophilus parainfluenzae T3T1*, previous research delineated that it was correlated with lung cancer, hence it may not be suitable to treat it as the especially potential biomarker of PC [37]. As for *Neisseria mucosa*, so far, no research displayed its percentage variation in any cancers, and *Fusobacterium periodonticum* also has no evidence of its correlation with cancers except for gastrointestinal tumors [38]. In Figure 2(c), *Megasphaera micronuciformis* performed a high LDA score, but it also appeared in the Figure 2(a), which means that no bacteria at the species level can specially be applied for identifying BPD patients from healthy individuals. In short, such performance suggested that the low abundance of *Neisseria mucosa*, and the high abundance of *Fusobacterium periodonticum*, stands out as potential specific risk factors for PC.

We are the first to try to comprehensively report the protective effect of *Neisseria mucosa* from PC. Oral diseases such as periodontal disease, caries, and tooth loss have been shown to be independent risk factors for the development of PC [39–41]. However, *Neisseria* in the oral cavity helps to prevent oral diseases [42]. *Neisseria* in the mouth can metabolize low-pH products such as lactic acid into weak acids and volatile acids, thereby protecting us from the caries [43]. In addition, the production of acid may contribute to the acidic and hypoxic microenvironment of the tumor, thereby improving the metastatic capacity [44, 45]. In existing studies, the abundance of *Neisseria elongata*, which belonged to *Neisseria* together with *Neisseria mucosa*, was significantly lower in the PC group than that in the HC group [18]. What we suspect is that the sharp decrease of *Neisseria* was usually accompanied by an increase in other pathogenic bacteria. In fact, these studies also described that Toll-like receptors recognize antigens of pathogenic bacteria and thereby modulate immune responses. In addition, inflammatory cytokines activate NF-*κ*B-associated cellular pathways and then regulate the expression of genes involved in inflammation, growth factors, and cell invasion molecules. NF-*κ*B is continuously activated in PC tissue cells. To sum up, the role of *Neisseria mucosa* in the oral cavity may be a protective factor for PC, but the association between *Neisseria* and PC needs further exploration.

In this study, we also first try to comprehensively report the pathogenetic effect of *Fusobacterium periodonticum* on the development of PC. *Fusobacterium nucleatum* has been shown to be involved in the development of colon cancer, and its concentration levels in the adenomas and cancer tissues were higher [46]. Moreover, studies have revealed that FadA, a virulence factor secreted by *Fusobacterium nucleatum*, is an important factor in carcinogenesis [47]. FadA binds to E-catenin, activates *β*-catenin, leads to upregulation of “proinflammatory factors, wnt signaling, and oncogenes,” and stimulates cell proliferation [48]. Actually, *Fusobacterium periodonticum* and *Fusobacterium nucleatum* are the only bacteria in the genus *Fusobacterium* that can secrete FadA virulence factors. In addition, oral bacteria can spread to the pancreas through blood circulation and biliary conduction [15]. A previous study has presented that the detection rate of *Fusobacterium* in PC tissues (283 cases) was 8.8%, and the presence of *Fusobacterium* was independently associated with poor prognosis of PC (*P* value < 0.05), suggesting that *Fusobacterium* may be a novel biomarker for predicting the prognosis of PC [49]. The case-control studies also support our above inference [36, 50]. Taken together, adhesion of the exogenous virulence factor FadA of the *Fusobacterium periodonticum* to host epithelial cells is most likely one of the molecular mechanisms which lead to PC.

Although the pathway we found in the current study is predicted based on the 16S sequence, which is not completely true, it can provide some clues for exploring the functions of these bacteria in the carcinogenicity and development of pancreatic cancer. In the pathway enrichment outcomes, totally fifty-two KEGG pathways were apparently different between the research groups (*P* value < 0.05). Eight KEGG pathways shown in Figure 3(a) were commonly changed in the PC and BPD groups compared to the HC group. It is meaningful that four pathways were related to glucose metabolism, which contains N-glycan biosynthesis, the synthesis of N-glycan biosynthesis, various types of N-glycan biosynthesis, lipopolysaccharide biosynthesis, and peptidoglycan biosynthesis. Obviously, glucose metabolism has a strong relationship with pancreatic diseases. For example, diabetes usually presented as a complication of chronic pancreatitis [51]. The occurrence and development of tumors are frequently accompanied by the changes of glycosylated proteins, and actually that there are significant changes in carbohydrate chains on the specific proteins of PC cell [52]. The predictive model based on N-glycan is also proved as an effective method for the diagnosis of PC [53]. Previous research has demonstrated that CA19.9 (carbohydrate antigen 19.9) synergizes with oncogene to promote the growth of PC [54]. In addition, among the forty specifically changed KEGG pathways in PC, eight pathways were upregulated and thirty-two pathways were downregulated. Generally speaking, the cell signaling pathway usually plays an important role in inflammation, immunity, cell proliferation, differentiation, and survival. The perturbation of the NOD-like receptor signaling pathway was confirmed to be significantly associated with PC [55], and this pathway also showed noticeable upregulation in our PC group. As bacteria cause the occurrence or growth of cancer by inducing an inflammatory microenvironment, the inflammatory factor in turn leads to cell proliferation, mutations, and oncogene activation [11]. Therefore, the alterations of oral bacteria may affect the development of PC through the natural bacterial antibacterial signaling pathway.

The changes in bacteria composition usually accompany the alterations of biological functions. We predicted the corresponding gene functions and metabolic pathways based on the 16S rDNA high-throughput sequencing results via the PICRUSt toolkit. Among the predicted results, five COG functions showed significant association with PC (*P* value < 0.05). As for RNA processing and modification, the existing study has proved that the RNA modification of the *METTL3* gene (m6A) promotes the development of PC and antichemotherapy/antiradiation therapy [56]. To intracellular trafficking, secretion, and vesicular transport, it is obvious that the secretion of insulin from pancreatic *β* cell is the vital function of the pancreas, and insulin dysregulation is a risk factor for pancreatic cancer [57]. With regard to inorganic ion transport and metabolism, the previous study showed us that the growth of malignant pancreatic tumors may be related to the metabolism of inorganic ion copper, which is essential to participate in the redox process in cells, and it is also a catalytic cofactor for various enzymes in organisms [58]. All of these reflected the specific association of PC and the above-mentioned biological functions.

## 5. Conclusion

In summary, we described the differences of salivary bacterium composition among the PC, BPD, and HC groups. The results in this study confirmed that oral bacterial community composition can be used for distinguishing PC from healthy people as well as the BPD patients. Lower levels of *Neisseria mucosa* and higher levels of *Fusobacterium periodonticum* from the oral cavity may be risk factors for the development of PC. However, more samples are needed to confirm the causal relationship between oral bacteria and PC. Conclusively, our findings supplement new knowledge to the medical problem of early diagnosis or prevention of PC.

## Data Availability

The raw data used to support the findings of this study were deposited into the NCBI Sequence Read Archive (SRA) database (Bioproject accession Number: SRP237984).

## Supplementary Materials

Supplementary Materials Table S1: clinical characteristics for all volunteers. Figure S1: bar plots depicted oral bacteria-alpha-diversity differences among the PC, BPD, and HC groups.

## References

[1] Bray F., Ferlay J., Soerjomataram I., Siegel R. L., Torre L. A., Jemal A. (2018). Global cancer statistics 2018: GLOBOCAN estimates of incidence and mortality worldwide for 36 cancers in 185 countries. *CA: a Cancer Journal for Clinicians*.

[2] Kleeff J., Korc M., Apte M. (2016). Pancreatic cancer. *Nature Reviews Disease Primers*.

[3] Siegel R. L., Miller K. D., Jemal A. (2019). Cancer statistics, 2019. *CA: A Cancer Journal for Clinicians*.

[4] De La Cruz M. S., Young A. P., Ruffin M. T. (2014). Diagnosis and management of pancreatic cancer. *American Family Physician*.

[5] Yeo C. J., Cameron J. L., Lillemoe K. D. (1995). Pancreaticoduodenectomy for cancer of the head of the pancreas. 201 patients. *Annals of Surgery*.

[6] Frena A. (2001). SPan-1 and exocrine pancreatic carcinoma. The clinical role of a new tumor marker. *The International Journal of Biological Markers*.

[7] Kim J. E., Lee K. T., Lee J. K., Paik S. W., Rhee J. C., Choi K. W. (2004). Clinical usefulness of carbohydrate antigen 19-9 as a screening test for pancreatic cancer in an asymptomatic population. *Journal of Gastroenterology and Hepatology*.

[8] Zhang Y., Yang J., Li H., Wu Y., Zhang H., Chen W. (2015). Tumor markers CA19-9, CA242 and CEA in the diagnosis of pancreatic cancer: a meta-analysis. *International Journal of Clinical and Experimental Medicine*.

[9] The Integrative HMP (iHMP) Research Network Consortium (2014). The Integrative Human Microbiome Project: dynamic analysis of microbiome-host omics profiles during periods of human health and disease. *Cell Host & Microbe*.

[10] Aas J. A., Paster B. J., Stokes L. N., Olsen I., Dewhirst F. E. (2005). Defining the normal bacterial flora of the oral cavity. *Journal of Clinical Microbiology*.

[11] Karpinski T. M. (2019). Role of oral microbiota in cancer development. *Microorganisms*.

[12] Ahn J., Segers S., Hayes R. B. (2012). Periodontal disease, Porphyromonas gingivalis serum antibody levels and orodigestive cancer mortality. *Carcinogenesis*.

[13] Castellarin M., Warren R. L., Freeman J. D. (2012). Fusobacterium nucleatum infection is prevalent in human colorectal carcinoma. *Genome Research*.

[14] Sethi V., Vitiello G. A., Saxena D., Miller G., Dudeja V. (2019). The role of the microbiome in immunologic development and its implication for pancreatic cancer immunotherapy. *Gastroenterology*.

[15] Brook I., Frazier E. H. (1996). Microbiological analysis of pancreatic abscess. *Clinical Infectious Diseases*.

[16] Meurman J. H. (2010). Oral microbiota and cancer. *Journal of Oral Microbiology*.

[17] Fan X., Fletcher E. M., Gibbons S. M., Bouvet M., Doran K. S., Kelley S. T. (2017). Human oral microbiome and prospective risk for pancreatic cancer: a population-based nested case-control study. *Gut*.

[18] Farrell J. J., Zhang L., Zhou H. (2012). Variations of oral microbiota are associated with pancreatic diseases including pancreatic cancer. *Gut*.

[19] Torres P. J., Fletcher E. M., Gibbons S. M., Bouvet M., Doran K. S., Kelley S. T. (2015). Characterization of the salivary microbiome in patients with pancreatic cancer. *PeerJ*.

[20] Magoc T., Salzberg S. L. (2011). FLASH: fast length adjustment of short reads to improve genome assemblies. *Bioinformatics*.

[21] Kuczynski J., Stombaugh J., Walters W. A., Gonzalez A., Caporaso J. G., Knight R. (2011). Using QIIME to analyze 16S rRNA gene sequences from microbial communities. *Current Protocols in Bioinformatics*.

[22] Cole J. R., Chai B., Marsh T. L. (2003). The Ribosomal Database Project (RDP-II): previewing a new autoaligner that allows regular updates and the new prokaryotic taxonomy. *Nucleic Acids Research*.

[23] Virtanen P., Gommers R., Oliphant T. E. (2020). SciPy 1.0: fundamental algorithms for scientific computing in Python. *Nature Methods*.

[24] Segata N., Izard J., Waldron L. (2011). Metagenomic biomarker discovery and explanation. *Genome Biology*.

[25] Langille M. G. I., Zaneveld J., Caporaso J. G. (2013). Predictive functional profiling of microbial communities using 16S rRNA marker gene sequences. *Nature Biotechnology*.

[26] Angiuoli S. V., Matalka M., Gussman A. (2011). CloVR: a virtual machine for automated and portable sequence analysis from the desktop using cloud computing. *BMC Bioinformatics*.

[27] Galperin M. Y., Makarova K. S., Wolf Y. I., Koonin E. V. (2015). Expanded microbial genome coverage and improved protein family annotation in the COG database. *Nucleic Acids Research*.

[28] Galvão-Moreira L. V., da Cruz M. C. F. N. (2016). Oral microbiome, periodontitis and risk of head and neck cancer. *Oral Oncology*.

[29] Mager D. L., Haffajee A. D., Devlin P. M., Norris C. M., Posner M. R., Goodson J. M. (2005). The salivary microbiota as a diagnostic indicator of oral cancer: a descriptive, non-randomized study of cancer-free and oral squamous cell carcinoma subjects. *Journal of Translational Medicine*.

[30] Sasaki M., Yamaura C., Ohara-Nemoto Y. (2005). Streptococcus anginosus infection in oral cancer and its infection route. *Oral Diseases*.

[31] Sakamoto H., Naito H., Ohta Y. (1999). Isolation of bacteria from cervical lymph nodes in patients with oral cancer. *Archives of Oral Biology*.

[32] Shiga K., Tateda M., Saijo S. (2001). Presence of Streptococcus infection in extra-oropharyngeal head and neck squamous cell carcinoma and its implication in carcinogenesis. *Oncology Reports*.

[33] Flanagan L., Schmid J., Ebert M. (2014). Fusobacterium nucleatum associates with stages of colorectal neoplasia development, colorectal cancer and disease outcome. *European Journal of Clinical Microbiology & Infectious Diseases*.

[34] Narikiyo M., Tanabe C., Yamada Y. (2004). Frequent and preferential infection of Treponema denticola, Streptococcus mitis, and Streptococcus anginosus in esophageal cancers. *Cancer Science*.

[35] Lu H., Ren Z., Li A. (2016). Deep sequencing reveals microbiota dysbiosis of tongue coat in patients with liver carcinoma. *Scientific Reports*.

[36] Lu H., Ren Z., Li A. (2019). Tongue coating microbiome data distinguish patients with pancreatic head cancer from healthy controls. *Journal of Oral Microbiology*.

[37] Kosikowska U., Biernasiuk A., Rybojad P., Los R., Malm A. (2016). Haemophilus parainfluenzae as a marker of the upper respiratory tract microbiota changes under the influence of preoperative prophylaxis with or without postoperative treatment in patients with lung cancer. *BMC Microbiology*.

[38] Saffarian A., Mulet C., Regnault B. (2019). Crypt- and mucosa-associated core microbiotas in humans and their alteration in colon cancer patients. *mBio*.

[39] Hujoel P. P., Drangsholt M., Spiekerman C., Weiss N. S. (2003). An exploration of the periodontitis-cancer association. *Annals of Epidemiology*.

[40] Michaud D. S., Joshipura K., Giovannucci E., Fuchs C. S. (2007). A prospective study of periodontal disease and pancreatic cancer in US male health professionals. *Journal of the National Cancer Institute*.

[41] Stolzenberg-Solomon R. Z., Dodd K. W., Blaser M. J., Virtamo J., Taylor P. R., Albanes D. (2003). Tooth loss, pancreatic cancer, and Helicobacter pylori. *The American Journal of Clinical Nutrition*.

[42] Liu G., Tang C. M., Exley R. M. (2015). Non-pathogenic Neisseria: members of an abundant, multi-habitat, diverse genus. *Microbiology*.

[43] Hoshino E., Araya A. (1980). Lactate degradation by polysaccharide-producing Neisseria isolated from human dental plaque. *Archives of Oral Biology*.

[44] Lunt S. J., Chaudary N., Hill R. P. (2009). The tumor microenvironment and metastatic disease. *Clinical & Experimental Metastasis*.

[45] Mazzio E. A., Smith B., Soliman K. F. (2010). Evaluation of endogenous acidic metabolic products associated with carbohydrate metabolism in tumor cells. *Cell Biology and Toxicology*.

[46] Rubinstein M. R., Wang X., Liu W., Hao Y., Cai G., Han Y. W. (2013). Fusobacterium nucleatum promotes colorectal carcinogenesis by modulating E-cadherin/*β*-catenin signaling via its FadA adhesin. *Cell Host & Microbe*.

[47] Han Y. W., Ikegami A., Rajanna C. (2005). Identification and characterization of a novel adhesin unique to oral fusobacteria. *Journal of Bacteriology*.

[48] Gholizadeh P., Eslami H., Kafil H. S. (2017). Carcinogenesis mechanisms of Fusobacterium nucleatum. *Biomedicine & Pharmacotherapy*.

[49] Mitsuhashi K., Nosho K., Sukawa Y. (2015). Association of Fusobacterium species in pancreatic cancer tissues with molecular features and prognosis. *Oncotarget*.

[50] del Castillo E., Meier R., Chung M. (2019). The microbiomes of pancreatic and duodenum tissue overlap and are highly subject specific but differ between pancreatic cancer and noncancer subjects. *Cancer Epidemiology, Biomarkers & Prevention*.

[51] Liu Y., Wang D., Guo H. L. (2019). Risk factors and nomogram for diabetes mellitus in idiopathic chronic pancreatitis. *Journal of Gastroenterology and Hepatology*.

[52] Yue T., Goldstein I. J., Hollingsworth M. A., Kaul K., Brand R. E., Haab B. B. (2009). The prevalence and nature of glycan alterations on specific proteins in pancreatic cancer patients revealed using antibody-lectin sandwich arrays. *Molecular & Cellular Proteomics*.

[53] Zhao Y. P., Zhou P. T., Ji W. P. (2017). Validation of N-glycan markers that improve the performance of CA19-9 in pancreatic cancer. *Clinical and Experimental Medicine*.

[54] Engle D. D., Tiriac H., Rivera K. D. (2019). The glycan CA19-9 promotes pancreatitis and pancreatic cancer in mice. *Science*.

[55] Miskiewicz A., Szparecki G., Durlik M., Rydzewska G., Ziobrowski I., Gorska R. (2015). The Q705K and F359L single-nucleotide polymorphisms of NOD-like receptor signaling pathway: association with chronic pancreatitis, pancreatic cancer, and periodontitis. *Archivum Immunologiae et Therapiae Experimentalis (Warsz)*.

[56] Taketo K., Konno M., Asai A. (2018). The epitranscriptome m6A writer METTL3 promotes chemo- and radioresistance in pancreatic cancer cells. *International Journal of Oncology*.

[57] Wolpin B. M., Bao Y., Qian Z. R. (2013). Hyperglycemia, insulin resistance, impaired pancreatic *β*-cell function, and risk of pancreatic cancer. *Journal of the National Cancer Institute*.

[58] Yu Z., Zhou R., Zhao Y. (2019). Blockage of SLC31A1-dependent copper absorption increases pancreatic cancer cell autophagy to resist cell death. *Cell Proliferation*.

