# Supplementary material for: Characterization of Oral Microbiome and Exploration of Potential Biomarkers in Patients with Pancreatic Cancer
Source: Biomed Res Int. 2020 Oct 31;2020:4712498. doi: 10.1155/2020/4712498 (PMC7652608; doi:10.1155/2020/4712498)
Supplement: Supplementary Materials — Table S1: clinical characteristics for all volunteers. Figure S1: bar plots depicted oral bacteria-alpha-diversity differences among the PC, BPD, and HC groups. [file 4712498.f1.docx]

***Supplementary Material***

**Table S1.** Clinical characteristics for all volunteers.

| Clinical Indexes | PC (N = 10) | BPD (N = 17) | HC (N = 10) |
| --- | --- | --- | --- |
| Age (years±SD) | 57.4±7.8 | 42.8±16.0 | 31.1±2.7 |
| Gender(Male/Female) | 6/4 | 10/7 | 6/4 |
| Smoking | 2 | 3 | 2 |
| Drinking | 2 | 3 | 1 |
| Tumor differentiation | I-II, III-IV (N = 5, 5) | NA | NA |


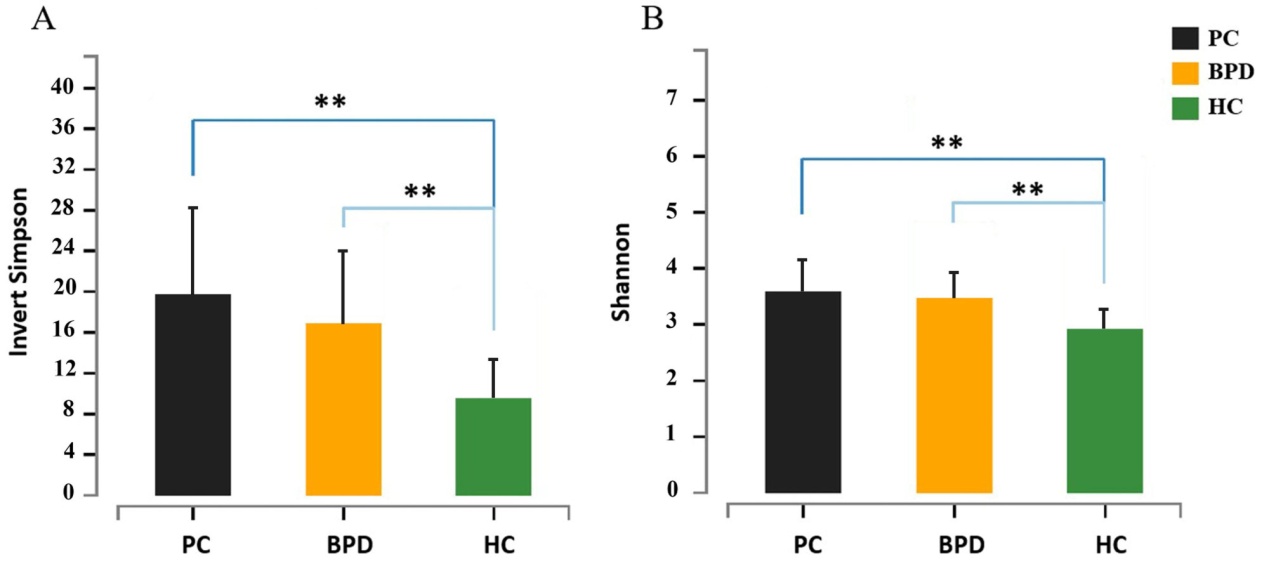


Figure S1:Bar plotsdepictedoral bacteriaalpha diversity differences according to the invert Simpson index (A) and Shannon index (B)among the PC, BPD and HC groups.*Note:* the symbol of ′*′ represents the P-value between groups is less than 0.05.
